# Condensates on the Move: Midbody Remnants as Large, Translation‐Competent Extracellular Vesicles

**DOI:** 10.1002/bies.70168

**Published:** 2026-08-04

**Authors:** Ahna R. Skop

**Affiliations:** ^1^ Laboratory of Genetics UW‐Madison Madison Wisconsin USA

**Keywords:** biomolecular condensates, cancer, cytokinesis, extracellular vesicles, intercellular communication, localized translation, midbody remnant, midbody, protein synthesis, stem cells

## Abstract

Recent work has expanded understanding of extracellular vesicle (EV) biology by identifying midbody remnants (MBRs) as large, translationally competent vesicles released during mitosis. MBRs contain ribosomes, mitochondria, translation factors, and selected mRNAs and small RNAs concentrated within a condensate‑like ribonucleoprotein core and can support protein synthesis after extracellular release. These features distinguish MBRs from more extensively studied exosomes and microvesicles yet also place them within a broader continuum of large EVs with organelle‑rich, cell‑like properties. This review summarizes current knowledge of MBR biogenesis, molecular organization, and translation competency; contrasts MBRs with canonical EVs and other large EVs; and discusses possible roles in development, tissue homeostasis, disease, and brain function. Particular emphasis is placed on outstanding mechanistic and physiological questions, including how MBR translation is regulated, which recipient cells interact with endogenous MBRs, and whether translation‑competent EVs offer practical advantages over existing EV platforms for therapeutic or biotechnological applications.

## Graphical abstract

Mitotic cells produce three EV types: exosomes and microvesicles (green/blue) delivering pre‐formed molecules, and midbody remnants (magenta) containing functional ribosomes and translation machinery within biomolecular condensates. Unlike passive cargo carriers, MBRs actively synthesize proteins after release, transforming from cellular debris to programmable protein factories.

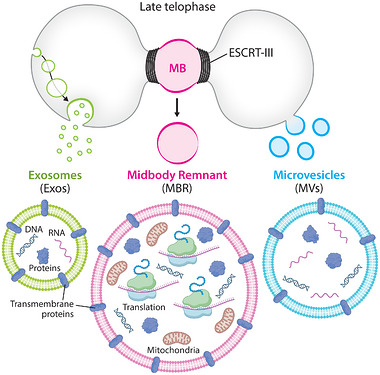

**Abbreviations**
Ago2: Argonaute 2
CFPS: cell‑free protein synthesis.
ESCRT: endosomal sorting complex required for transport
EV: extracellular vesicle
LEV: large extracellular vesicle
lncRNA: long non‑coding RNA;
MB: midbody
MBR: midbody remnant
miRNA: microRNA
mRNA: messenger RNA
RNA: ribonucleic acid
RNAi: RNA interference
RNP: ribonucleoprotein
rRNA: ribosomal RNA
tRNA: transfer RNA

## 1 Introduction

Extracellular vesicles (EVs) are a heterogeneous group of membrane‐bound structures released by nearly all cell types and are commonly classified by size, biogenesis, and molecular composition. This broad category includes exosomes, microvesicles, large EVs, and midbody remnants (MBRs), a mitotically derived EV population released during the final stages of cytokinesis [1, 2, 3, 4, 5, 6]. Once regarded mainly as post‐mitotic debris, MBRs are now recognized as bona fide large EVs with distinctive biogenesis, composition, and behavior. Recent studies show that MBRs are enriched in cytokinetic proteins, ribosomes, mitochondria, translation factors, and localized mRNAs and small RNAs, distinguishing them from more extensively studied EV subclasses [1, 2, 7, 8]. The finding that standard EV isolation workflows can also recover MBRs further suggests that mitotic EVs may have been included in prior EV preparations without being explicitly identified [1, 7].

EVs are broadly understood to mediate intercellular communication by transferring proteins, lipids, and multiple RNA species to recipient cells, contributing to roles in immune regulation, tissue repair, development, and disease [3, 9, 10, 11]. Recent work has added another layer to this framework by showing that MBRs can support protein synthesis after release [2]. These observations suggest that at least a subset of the large EVs may be biosynthetically active rather than functioning only as carriers of preassembled cargo. This review therefore examines MBRs within the broader EV field, with emphasis on their biogenesis, molecular organization, possible functional significance, and the questions that remain unresolved.

## 2 The Midbody Remnant: An Unconventional EV

### 2.1 Origin and Definition

During the final stages of cell division, the midbody (MB) forms between nascent daughter cells and coordinates abscission. The MB arises from the spindle midzone and contains structurally distinct regions, including the midbody ring, the central core, and the flanking arms. The MB matrix, now called the MB‐granule [2, 7], is an electron‐dense region enriched in proteins and RNAs involved in cytokinesis and, in some cases, cell fate‐associated pathways [1, 7, 12]. Recent work supports the view that the MB and the resulting MBR contain a phase‐separated ribonucleoprotein‐rich compartment that helps organize specific molecular components. Following completion of cytokinesis, the MBR can be released extracellularly and later taken up, retained, or degraded by surrounding cells [7, 13, 14]. In contrast to exosomes and microvesicles, whose biogenesis is linked to endosomal pathways or plasma membrane shedding [15, 16, 17], MBRs arise specifically from cytokinesis. Proteomic studies indicate that MBRs contain cytokinetic proteins, translation‐associated factors, RNA‐binding proteins, and mitochondrial components [12, 18, 19, 20]. These features support classifying MBRs as a distinct class of large EVs, although how broadly these properties generalize across cell types and physiological settings remains an active area of investigation [6, 7, 16].

### 2.2 Possible Functions Before Abscission

Most discussion of MBRs centers on events after release, but the midbody itself can also have functions before abscission is completed. In some systems, including early embryos, stable midbodies persist long enough to contribute to polarity, tissue organization, or signaling before extracellular release occurs [7, 21, 22, 23, 24, 25]. These observations raise the possibility that RNA localization and condensate‐like organization at the midbody first support cell‐intrinsic or bridge‐associated functions during cytokinesis, with extracellular MBR activity representing a subsequent extension of that biology. Considering pre‐abscission and post‐abscission functions together may therefore provide a more complete framework for understanding midbody‐derived signaling structures [7, 26].

### 2.3 Differences Between Translation‐Competent and Canonical EVs

Canonical EVs such as exosomes and microvesicles are well established as carriers of proteins, lipids, and RNAs that influence recipient‐cell behavior after uptake or surface interaction [15, 17, 27]. In most cases, however, any transferred mRNAs are presumed to rely on the recipient cell's translation machinery for protein production [11, 28, 29]. Although ribosomal proteins can be detected in some EV preparations [30, 31], the evidence for sustained autonomous translation in canonical EV populations has not been observed [1]. At the same time, canonical EVs are not inert particles: they exhibit regulated cargo selection, can trigger strong signaling responses, and may contain components derived from biomolecular condensates involved in RNA sorting [30, 31, 32].

MBRs differ from these more well‐studied EV classes in several respects. They are larger, arise specifically during mitosis, and contain concentrated translation‐associated machinery together with distinct RNA cargo. The MBR core includes a ribonucleoprotein‐rich compartment with properties consistent with condensate organization called the MB‐granule, and newly released MBRs can support protein synthesis after release [1, 2]. These observations support the idea that MBRs are a translation‐competent EV subtype, but the degree to which this property is maintained in different physiological contexts and maturation states remains to be defined.

**FIGURE 1:**
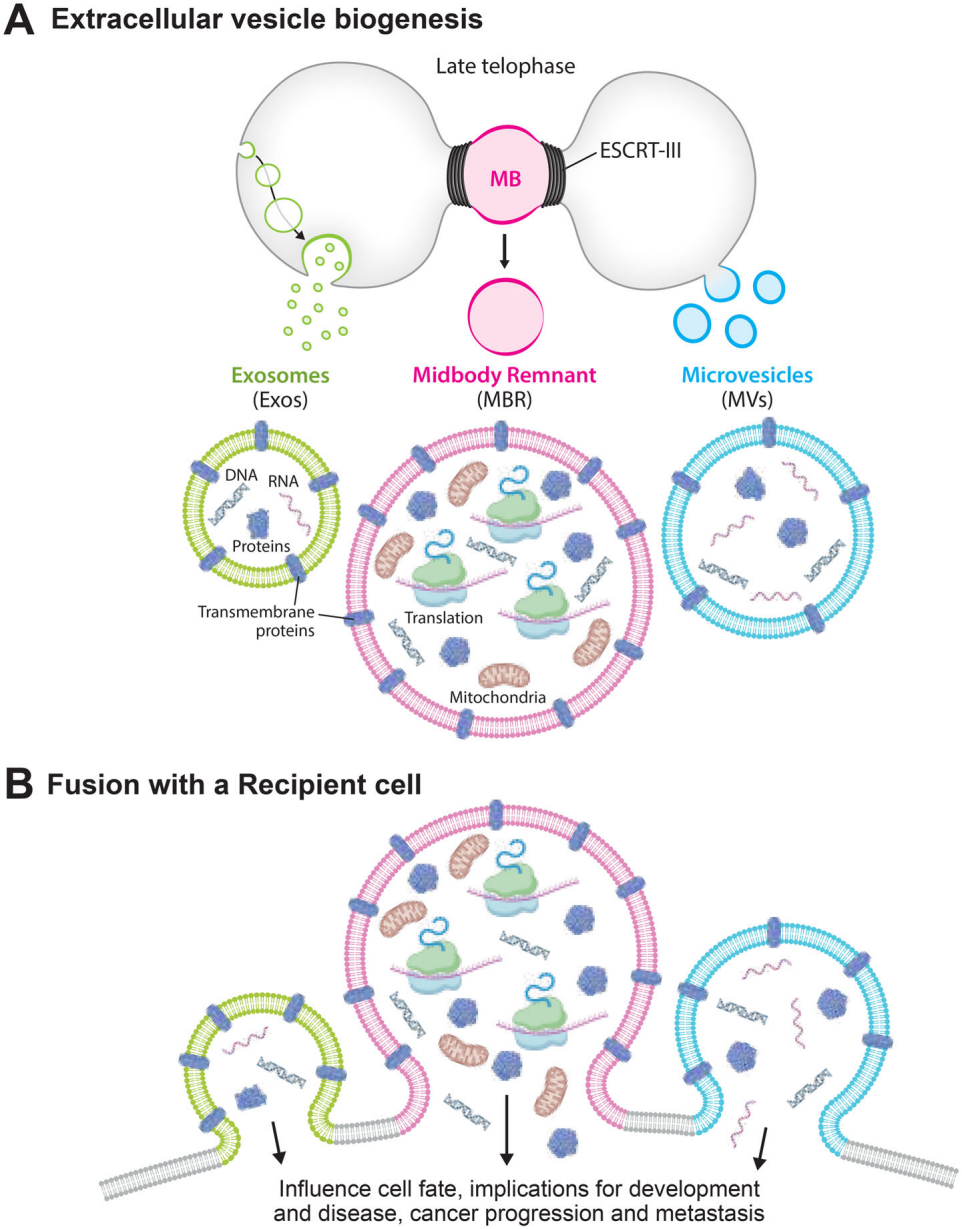
**Model for extracellular vesicle biogenesis during mitosis and fusion with a recipient cell**. (A) EV biogenesis in mitotic cells can produce exosomes, midbody remnants, and microvesicles. Exosomes and microvesicles contain RNA, DNA, and proteins, whereas MBRs also contain distinct translation machinery, mitochondria, and functional ribosomes. (B) Fusion of exosomes, MBRs, and microvesicles with recipient cells has been associated with effects on cell fate, development, disease, and cancer progression, although the relative contributions of each EV class remain to be clarified. Representative images of small, medium, and large EVs, including MBRs, have been reported previously (please see Patel et al. [1]).

Beyond MBRs, several additional large EV populations have been reported to display partial functional autonomy. Blebbisomes, migrasomes, amphiectosomes, exophers, and large oncosomes can carry organelles or complex RNA‐protein cargo and, in some cases, exhibit behaviors suggesting cell‐like activity [3, 17, 33, 34, 35, 36, 37, 38, 39, 40, 41]. Notably, blebbisomes act as cell‐autonomous mobile communication hubs that can both release and internalize smaller extracellular vesicles, providing a clear example of autonomous EV trafficking in the extracellular space. The extent to which these types of large vesicles support translation or other autonomous functions remains unresolved, but their existence argues against a simple active‐versus‐passive distinction across EV classes. Instead, current evidence supports a broader continuum in which different EV populations vary in their degree of functional complexity, biosynthetic capacity, and dependence on recipient cells. (Figure 1)

### 2.4 Biological Implications

The molecular organization of MBRs suggests that they may retain a degree of functional autonomy after release. By packaging selected RNAs, RBPs, and mitochondria together with translation‐associated factors inside a membrane‐enclosed compartment, MBRs may be able to support localized protein production [2, 7]. This differs from many EV models that emphasize delivery of preformed cargo alone, while also inviting comparison with other large EVs that may share related properties [11, 16, 34, 42]. At present, however, the downstream consequences of MBR translation for recipient cells and tissues are still not well defined, and direct evidence for sustained functional impact in vivo remains limited. (Tables 1 and 2)

**TABLE 1:** **Comparison of canonical extracellular vesicles and translation‐competent extracellular vesicles (MBRs)**. The table summarizes distinguishing features of these EV classes, including size, biogenesis, cargo composition, translation capacity, and possible biological or translational relevance.

**Feature**	**Canonical EVs (Exosomes, Microvesicles)**	**Translation‐Competent EVs (MBRs)**
**Size**	Canonical EVs (<600 nm), Exosomes (30–150 nm), Microvesicles (100–1,000 nm)	Larger (800nm–2.0 µm; correlates with cell size)
**EV classification**	Classified increasingly by size: small EVs (sEV, <200 nm) and medium/large EVs (m/lEV, >200 nm), not solely by origin/markers	Distinct classification based on biogenesis, MKLP1/KIF23, and translation competency; complements size‐based EV classification
**Population Heterogeneity**	Wide range in size/cargo across populations	MBRS typically uniform, size correlates with cell size
**Biogenesis**	Endosomal sorting (exosomes) or plasma membrane budding (microvesicles)	Cytokinetic abscission during cell division
**Molecular Cargo**	Pre‐formed proteins, lipids, small regulatory RNAs (miRNA, tRNA fragments), incomplete and some full‐length mRNAs	Ribosomal subunits, translation factors, mRNAs, miRNAs, lncRNAs (developmental, fate‐related), mitochondria, proteins
**Translation Machinery**	Largely absent in functional form; occasional ribosomal proteins detected but no robust evidence of active ribosomes	Complete set present (ribosomes, eIFs, eEFs, tRNA synthetases, cofactors); capable of active translation
**Markers and Isolation**	Common markers (CD9, CD63, CD81) overlap among EV types; no exclusive markers	Specific markers like MKLP1/KIF23 distinguish MBRs from other EVs
**RNA granule properties**	YBX1 condensates present in exosomes; stress granule and P‐body components detected	Exhibits hexanediol sensitivity; MKLP1/KIF23 is a unique phase‐separated kinesin (Kinesin 6 family), YBX3 enriched in MBRs
**Biochemical Activity**	Largely biologically inert after release; no evidence of autonomous translation	Autonomous, robust protein synthesis before and after detachment from newly formed daughter cells
**Mechanism of Action**	Courier: delivers cargo for recipient cell decoding and response	Active: executes protein synthesis “on demand” locally, can deliver cargo to recipient cells
**Regulatory Proteins**	Cargo‐sorting adaptors (e.g., ALIX); markers like CD9, CD63, CD81 overlap widely	Regulation by MKLP1 (KIF23), ARC, ESCRT‐III, and unique cytokinetic factors; MKLP1 is a specific marker
**Impact on Recipient**	Dependent on recipient cell's translation and signaling machinery	Can potentially alter local proteome and cellular microenvironment through local translation
**Signaling Modality**	Passive, typically triggers or modulates responses through delivered contents	Dynamic, potentially enables localized, stimulus‐responsive protein synthesis
**Isolation Challenges**	Marker overlap with MBRs hinders pure isolation	MKLP1/KIF23 marker enables specific isolation
**Therapeutic Utility**	Diagnostic, drug delivery applications	Potential as biomarkers and active delivery agent with biogenesis in mitosis

**TABLE 2:** **Midbody remnants within the spectrum of large extracellular vesicles**. This table situates midbody remnants (MBRs) within a broader spectrum of large extracellular vesicles, highlighting that while MBRs currently provide the strongest evidence for translation‐competent, condensate‐rich architecture, other large EV classes such as blebbisomes, migrasomes, amphiectosomes, exophers, and large oncosomes also exhibit complex cargo and behaviors that suggest varying degrees of functional autonomy, underscoring a continuum rather than a strict active–passive dichotomy across EV populations.

Large EV class	Distinguishing features	Evidence and open questions
Midbody remnants (MBRs)	Mitotically derived large EVs released from the midbody after cytokinesis; enriched in cytokinetic proteins, ribosomes, mitochondria, translation factors, and selected RNAs organized around a condensate‐like core.	Experimental data support translation competency and condensate organization, but the duration, regulation, and in vivo consequences of this activity remain incompletely defined; physiological roles and tissue‐specific relevance are still under investigation.
Blebbisomes	Large, organelle‐rich vesicles generated by blebbing‐related mechanisms; can contain mitochondria and complex cytoplasmic cargo and display cell‐like behaviors, including motility and vesicle uptake.	Suggest partial functional autonomy, but it is not yet known whether they support sustained protein synthesis; their biosynthetic capacity and relation to other large EVs remain to be clarified.
Migrasomes	Large EVs formed at retraction fibers during cell migration; carry cytoplasmic and membrane cargo and participate in intercellular signaling.	Show regulated formation and signaling activity, yet direct evidence for autonomous translation is lacking; their position on a spectrum of EV functional complexity is still being defined.
Amphiectosomes	Large vesicular structures arising from en bloc release of amphisome‐derived compartments, with mixed endosomal and cytoplasmic contents.	Reflect complex biogenesis and cargo heterogeneity, but potential for translation or other autonomous biosynthetic functions has not been established; detailed mechanistic and functional studies are needed.
Exophers	Stress‐associated, large vesicles that can carry organelles, aggregated proteins, and RNAs, particularly described in neural and proteotoxic settings.	Implicated in cellular quality control and stress responses; whether they exhibit translation competency or broader biosynthetic autonomy remains an open question.
Large oncosomes	Large membrane‐derived vesicles released by tumor cells; carry proteins, lipids, nucleic acids, and organelle‐derived components and are associated with cancer progression.	Clearly participate in pathological signaling and remodeling of tumor microenvironments, but there is no direct evidence for sustained autonomous translation; best viewed as complex signaling carriers whose biosynthetic potential is still uncertain.

### 2.5 Evolutionary Perspectives

Translation‑competent EVs could, in principle, provide selective advantages by allowing donor cells to transfer not only molecular cargo but also some capacity for local protein production [1, 6, 43]. Such a system might be useful in settings that require spatially restricted, rapid, or transient responses, including development, regeneration, and stress adaptation [43, 44, 45, 46, 47]. Similar logic underlies well‑known examples of localized translation in neurons and other polarized biological systems, although those processes do not necessarily involve vesicle‑mediated transfer between cells [48, 49, 50]. For MBRs, these evolutionary ideas remain speculative and are best viewed as a framework for testable hypotheses about when and why extracellular translation might emerge and persist in particular cellular contexts [42, 51, 52].

## 3 Mechanistic Insights Into Extracellular Vesicle‐Mediated Translation

### 3.1 Maintenance of Translational Activity Post‐Release

A central question raised by MBR biology is how translational activity is maintained after vesicle release. Current evidence indicates that MBRs contain ribosomes, mitochondria, translation factors, ribosomal RNAs, and selected messenger RNAs, thereby assembling core components needed for protein synthesis [2, 12, 18, 53]. The surrounding membrane is likely to protect these molecules from extracellular degradation, while the condensate organization of the MBR core, the MB‐granule, may help maintain their local concentration and functional coupling. Existing studies further suggest that translation begins during late cytokinesis and can continue after extracellular release, although the duration, regulation, and physiological consequences of this activity remain limited [1, 2, 7]. It also remains important to distinguish that translation initiates before release from translation sustained in fully extracellular MBRs, as resolving that point will be central to defining their true biosynthetic autonomy.

## 4 Biological Implications of Translation‐Competent Extracellular Vesicles

### 4.1 Intercellular Communication

If MBRs deliver both messenger RNAs, small RNAs, and translation machinery to recipient cells, they could extend extracellular communication beyond simple cargo transfer [1, 2, 6, 16, 54]. In that scenario, recipient cells or nearby tissue environments might gain access to localized protein production without relying solely on transcriptional responses in the recipient cell nucleus [27, 55, 56, 57]. This model remains under active investigation, but it provides a plausible framework for how MBRs could alter nearby cellular states in a temporally and spatially restricted manner. At present, however, the identity of the most relevant recipient cells, the routes of MBR uptake, and the extent of functional protein synthesis after uptake remain unresolved.

### 4.2 Role of RNA Interference

There is also interest in whether MBRs carry components of the RNA interference (RNAi) machinery. Ago2 has been observed at the midbody during cytokinesis [58], and Argonaute proteins have been reported in some EV preparations, although the literature remains technically and conceptually mixed on this point [59, 60]. Conventional sizing and analysis approaches, including nanoparticle tracking instruments with upper detection limits around 1.0 µm, may underrepresent larger EV populations such as MBRs that can reach 1–2 µm in diameter [61], potentially leading to underestimation of Ago2‐containing vesicles in standard preparations. If RNAi‐associated factors were transferred alongside mRNAs and translation machinery, they could, in principle, modulate gene expression in recipient cells at both translational and post‐transcriptional levels, thereby expanding the potential influence of MBR‐mediated communication. Such a dual capacity for protein synthesis and gene regulation could be particularly relevant during development, tissue repair, or disease progression, where precise temporal and spatial control of gene expression is crucial. These considerations make it reasonable to ask whether MBRs might couple translation‐associated cargo with post‐transcriptional regulatory factors, but direct evidence for a functional RNAi program operating within extracellular MBRs is still lacking, and any such role should currently be regarded as a hypothesis rather than an established property.

### 4.3 Horizontal Gene Transfer and RNA Inheritance

MBRs also raise questions about non‐genetic transfer of information between cells [62, 63, 64]. By moving messenger and small RNAs together with translation‐associated machinery, they could in principle promote transient expression of proteins not otherwise produced by recipient cells [56, 65]. If regulatory RNAs or RNAi‐associated factors are also transferred, the scope of that influence could extend beyond protein production alone [27, 43, 64]. At present, however, these possibilities remain conceptual extensions of the available data and should be interpreted cautiously, particularly because most direct evidence for EV‐mediated horizontal transfer comes from broader EV systems rather than MBR‐specific studies. Defining whether endogenous MBRs can mediate such effects in vivo will require direct experimental validation in physiologically relevant settings.

### 4.4 Development and Tissue Homeostasis

MBRs have the potential to influence development and tissue homeostasis, but direct in vivo evidence for such roles remains limited. Because they contain localized RNAs and translation machinery [1, 2], it is plausible that they could contribute to spatially restricted signaling in stem cell niches, regenerative environments, or other contexts in which local protein production may be advantageous [66, 67, 68]. For example, if MBRs deliver or translate regulatory factors within target microenvironments, they could help modulate cell‑state transitions or local signaling landscapes [69, 70], a possibility that currently rests on inference from cargo composition and broader EV literature rather than direct observation of endogenous MBRs in developing tissues.

Similarly, MBRs might participate in tissue repair or remodeling by supplying localized biosynthetic activity in extracellular space. This idea is conceptually attractive because many regenerative processes depend on tightly controlled spatial and temporal signaling [2, 19, 70, 71], and other EVs have been implicated in wound healing and organ repair [72, 73, 74, 75]. However, most current data relevant to these scenarios come from bulk EV preparations or non‑MBR EV types, and future studies that track endogenous MBRs in developmental and regenerative systems will be required to determine whether these possibilities are realized physiologically.

### 4.5 Disease and Pathology

MBRs may also be relevant to disease, particularly in cancer and other settings in which extracellular signaling influences cell behavior [1, 2, 76, 77, 78]. Their translation competency raises the possibility that they could contribute to local synthesis of proteins that affect proliferation, stress responses, or interactions within the tumor microenvironment [78, 79, 80, 81, 82, 83]. More broadly, comparisons with other EVs suggest that MBRs could participate in pathological communication networks, but direct evidence specifically assigning these functions to MBRs remains limited. It is therefore important to distinguish between the well‐established disease relevance of extracellular vesicles as a broad class and the still‐emerging question of whether MBRs make unique contributions within those contexts. At present, disease‐related roles for MBRs are best framed as important areas for investigation rather than established mechanisms.

### 4.6 Brain Function

MBRs could contribute to brain function if their translation competency operates in neural tissue in vivo, but this possibility has not yet been directly tested. In principle, their ability to support local protein synthesis outside the parent cell could make them relevant to spatially restricted signaling processes in neurons or glia [2, 7]. This idea is consistent with the broader importance of local translation in the nervous system [50, 84, 85], although no direct evidence currently demonstrates that MBRs play a defined role in synaptic plasticity, learning, repair, or degeneration. Other large EV populations present in neural contexts may also contribute to related activities, making comparative in vivo studies especially important [86, 87, 88]. For now, possible neural roles for MBRs should be considered biologically plausible but unproven.

MBRs may also be worth considering in the context of neurodegeneration and neural repair [89, 90, 91]. If they transfer selected RNAs, proteins, or translation‐associated machinery in the brain, they could in principle influence local proteostasis or stress responses [90, 92]. At the same time, such possibilities remain speculative and should be clearly separated from the well‐established evidence for local translation within neurons and glia themselves. A major unresolved issue is whether MBRs are generated, persist, and are taken up at meaningful levels in intact neural tissue under physiological or pathological conditions.

### 4.7 Therapeutic and Technological Opportunities

The identification of translation‑competent MBRs suggests potential therapeutic and biotechnological applications, although these remain largely prospective. In principle, vesicles that combine selected RNAs with translation‑associated machinery could be used to achieve localized protein production in target tissues [93, 94, 95], complementing existing EV‑based delivery strategies and cell‑free protein synthesis systems. This concept may be relevant to drug delivery, regenerative medicine, and synthetic biology, particularly if MBR‑like platforms can be engineered in a controlled and scalable way [93, 96, 97]. At the same time, it is not yet clear whether MBRs offer practical advantages over better‑characterized EV platforms, and challenges related to stability, targeting, manufacturability, and immunogenicity will need to be addressed before translational development is prioritized.

MBRs may also provide a useful natural model for compartmentalized translation. Their organization could inform design principles for vesicle‑based cell‑free systems or programmable biosynthetic platforms that decouple protein production from living cells [96, 98, 99]. For now, however, the main value of MBRs in this area is conceptual: insights from their condensate‑rich architecture and translation competency may guide the design of synthetic vesicles, even if MBRs themselves are not ultimately used as therapeutic vehicles.

### 4.8 Outstanding Questions and Future Directions

Many basic questions remain unresolved. It is still unclear how translation is regulated within extracellular MBRs, how cargo is selected and organized, how the condensate properties change after release, and how long the biosynthetic activity can be maintained under physiological conditions. It is also not yet known which recipient cells interact with endogenous MBRs in vivo, whether MBR functions differ by tissue or cell type, or how their activities compare directly with those of other large EV populations. Progress in these areas will require improved isolation strategies for large EVs, single‐vesicle analysis, higher‐resolution live imaging, and comparative studies across EV subclasses. In particular, experiments that selectively track and manipulate endogenous MBRs in vivo will be essential for determining whether the functions proposed here are physiologically relevant.

## 5 Conclusion

MBRs expand the current framework for extracellular vesicle biology by highlighting a mitotically derived EV population with evidence of translation competency and biomolecular condensate organization [1, 2, 53]. Their composition and behavior suggest that some EVs may do more than transfer preformed cargo, although the physiological significance of this capability remains to be established. The field now faces the task of determining when, where, and to what extent MBRs contribute to intercellular communication in vivo, and whether they differ functionally from other small or large EV populations with organelle‐rich or cell‐like features. Clarifying these questions will be essential before strong conclusions can be drawn about the roles of the diversity of EVs in development, disease, or therapeutic applications.

## Conflicts of Interest

A.R.S. is a co‐founder, equity holder, and officer of aMBR Genomics, Inc., a company developing technologies related to midbody remnants. This financial interest has been disclosed to the University of Wisconsin‐Madison and is managed in accordance with institutional policies.

## Data Availability

Data sharing not applicable to this article as no datasets were generated or analyzed during the current study
